# Association of Multiple Glycemic Parameters at Hospital Admission with Mortality and Short-Term Outcomes in Acutely Poisoned Patients

**DOI:** 10.3390/diagnostics11020361

**Published:** 2021-02-20

**Authors:** Catalina Lionte, Cristina Bologa, Inga Agafiti, Victorita Sorodoc, Ovidiu Rusalim Petris, Elisabeta Jaba, Laurentiu Sorodoc

**Affiliations:** 1 Internal Medicine and Clinical Toxicology Department, Faculty of Medicine, “Grigore T. Popa” University of Medicine and Pharmacy, 700115 Iasi, Romania; crisbologa@yahoo.com (C.B.); laurentiu.sorodoc@gmail.com (L.S.); 2 2nd Internal Medicine Clinic, “Sf. Spiridon” Emergency Clinic County Hospital, 700111 Iasi, Romania; agafiti.inga@gmail.com (I.A.); ovidiupetris@yahoo.com (O.R.P.); 3 Internal Medicine and Nursing Department, Faculty of Medicine, “Grigore T. Popa” University of Medicine and Pharmacy, 700115 Iasi, Romania; 4 Statistics Department, FEEA, “Alexandru Ioan Cuza” University, 700506 Iasi, Romania; elisjaba@gmail.com

**Keywords:** glycemia, mean glucose level, glucose variability, outcomes, poisoning, xenobiotics

## Abstract

The prognostic value of multiple glycemic parameters in poisoned patients was never assessed. We aim to explore the effects of glucose variability on short-term outcomes in nondiabetic and diabetic patients acutely poisoned with undifferentiated xenobiotics. We performed a prospective observational study in a tertiary center for toxicology in northeastern Romania. Over the course of 3 years, we included 1076 adults, older than 18 years, admitted for acute poisoning with a xenobiotic. The mortality rate was 4.1%. The admission blood glucose level (BGL) predicted mortality (OR 1.015, 95% CI 1.011–1.019, *p* < 0.001) and complications (OR 1.005, 95% CI 1.001–1.009, *p* 0.02). The mean glucose level (MGL) after admission (OR 1.007, 95% CI 1.000–1.013, *p* 0.034) and coefficient of glucose variability (CV) were predictive for complications (OR 40.58, 95% CI 1.35–1220.52, *p* 0.033), using the same multivariable model. The receiver operating characteristic curve (ROC) analysis revealed that BGL had good predictive value for in-hospital mortality (area under the curve (AUC) = 0.744, 95% CI = 0.648–0.841, *p* < 0.001), and complications (AUC = 0.618, 95% CI = 0.584–0.653, *p* < 0.001). In patients acutely poisoned with xenobiotics, the BGL, MGL and CV can be useful as mortality and short-outcome predictors.

## 1. Introduction

Hyper- and hypoglycemia are a common problem in hospitalized patients with or without a history of diabetes mellitus (DM) [1,2]. Hyperglycemia proved to be associated with increased morbidity, mortality and poor outcomes in patients with an acute illness, such as coronary syndromes, pneumonia, or exacerbation of a chronic obstructive pulmonary disease [3,4,5]. Hyperglycemia might exert an even more deleterious effect on those patients without DM than among patients with DM during acute illness [6]. 

Increased glucose variability was associated with longer hospitalization and mortality in both nondiabetic and diabetic patients, as well as with in-hospital complications following surgery [7,8]. Hyperglycemia is not a common feature of overdose [9]. There is a wide range of studies about hyperglycemia in both medical, surgical, and intensive care unit (ICU) patients [9,10,11,12,13]. To our best knowledge, hyperglycemia due to poisoning has been studied only in acute intoxication with methanol and pesticides where it proved to be a prognostic factor for lethality [14,15,16,17]. We have not found any study on non-diabetic and diabetic patients following acute poisoning with undifferentiated xenobiotics to show multiple glycemic parameters with respect to poisoning severity score and outcome. In particular, the predictive value of multiple glycemic parameters for in-hospital mortality is unclear. 

Therefore, the present study aims to assess if admission blood glucose level (BGL) and glucose variability are associated with the short-term outcomes in both diabetic and nondiabetic acutely poisoned patients admitted within 12 h of exposure to a medical or ICU ward. Thus, the optimal care of critically poisoned patients with hyperglycemia can be improved to decrease the mortality rate among these patients.

## 2. Materials and Methods

### 2.1. Study Population

From July 2017 to June 2020, we prospectively enrolled consecutive patients with acute poisoning presented to the Emergency Department (ED) of an urban tertiary hospital with over 100,000 ED visits annually, which is a referral center for clinical toxicology in northeastern Romania. Patients eligible for enrollment were adults older than 18 years, with a diagnosis of acute poisoning within 12 h of exposure to a xenobiotic, as the primary reason for admission, hospitalized in a medical or ICU ward. Patients were excluded if there was a lack of a signed informed consent, were younger than 18 years, had received intravenous dextrose solution, glucocorticoids, catecholamines, glucagon or diazoxide before sampling [18,19]. In addition, patients with another emergency associated (e.g., trauma, burns), or incomplete data were excluded. 

### 2.2. Baseline Data Collection

The following data were collected: baseline characteristics, vital signs, mental status, underlying diseases, the Charlson comorbidity index (CCI) calculated according to the scoring system established by Charlson et al. [20], the xenobiotic involved, time from exposure, the intent of the poisoning (self-harm or accident), co-ingestions, laboratory test results upon presentation in the ED and during hospitalization, the poisoning severity score (PSS) grading as (0) none, (1) minor, (2) moderate, (3) severe, and (4) fatal [21,22], the complications, ICU admission days, and in-hospital outcomes. Based on their CCI score, patients were divided into three groups: mild, with CCI scores of 1–2; moderate, with CCI scores of 3–4; severe, with CCI scores ≥ 5. The patients were monitored during hospitalization. Blood glucose samples were obtained on ED admission and subsequently every 3 h for 12 h; after that, upon indication of the attending physician. In cases of hypoglycemia, the glucose level was checked every hour by glucometer until the normal blood glucose levels were obtained. The BGL upon ED admission was measured using the ARCHITECT c16000 clinical chemistry analyzer (Abbott Laboratories, Abbott Park, IL, USA). The BGL upon admission was divided into hypoglycemia (<70 mg/dL), normoglycemia (70–100 mg/dL), impaired glucose level (101–139 mg/dL), and hyperglycemia (>140 mg/dL) ranges, based on the guidelines’ threshold for in-hospital hyperglycemia [23,24,25]. We collected all glucose values measured for every patient and calculated the mean glucose level (MGL) during hospitalization and the SD. The mean amplitude of glycemic excursions (MAGE) was defined as the mean of the absolute values of any delta glucose from consecutive measurements that were higher than the SD of the entire set of glucose values [26]. The mean absolute glucose (MAG) change per patient per hour, defined as the sum of the absolute value of all glucose changes during the time of observation, divided by the total time of observation (mg/dL/h) was calculated [10]. Coefficient of variation (CV) of glucose (SD/MGL, [%]) was derived for each patient. 

### 2.3. Outcomes and Definitions

The primary outcome was in-hospital mortality. Secondary endpoints were time spent in the ICU and in-hospital complications related to the poisoning, such as acute liver injury and acute kidney injury, defined according to the guidelines [27,28]. We defined the patients discharged without any complication as result of the poisoning as having a good outcome, a moderate outcome was for the patients with in-hospital complications and a poor outcome was for the patients deceased during hospitalization. 

### 2.4. Statistical Analysis

Numerical variables are presented as mean ± SD for normally distributed continuous data, median with interquartile range for non-normally distributed continuous data, or frequency for categorical variables. Independent sample t test or Mann–Whitney U test, as appropriate were used to identify significant differences between the outcome groups defined. The Chi-square test and Cochrane’s statistic for categorical variables were used to perform univariate analysis. All variables found to be significant in the univariate analyses for the outcomes were subjected to a multivariate logistic regression analysis. Risk was expressed as odds ratios (ORs) with confidence intervals (CIs). The receiver operating characteristic curve (ROC) was used as a measure of diagnostic performance, to validate the discriminatory power of the model predictive variables. All tests were two-tailed, and a *p*-value < 0.05 was considered statistically significant. Statistical analyses were performed with SPSS (version 22.0; SPSS, Inc., Chicago, IL, USA). 

## 3. Results

### 3.1. Baseline Characteristics

**Figure 1:**
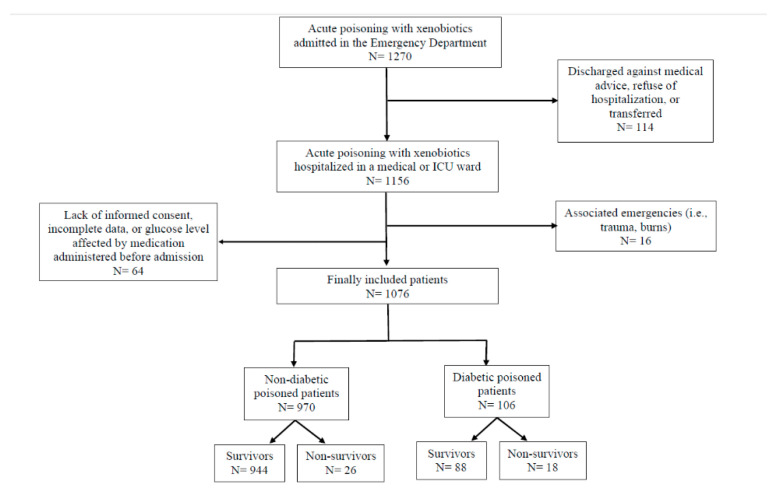
Study flow diagram.

A total of 1076 patients (51.9% females) were enrolled (Figure 1). Median age was 45 years (range 18–98 years), 74.3% had intentional poisoning, 11.6% were overweight, and 11.5% were obese. The median time from poison exposure was 3 h (ranged from 30 min to 12 h). The main categories of poisons involved were prescription drugs 31.5%, pesticides 10.3%, toxic alcohols and chemicals 8.6%, caustic agents 7.7%, toxic gases 5.5%. A combination of poisons were recorded in 26.2% patients. Over-the-counter medication, illicit drugs, or plant toxins represented the rest of the cases (10.1%). Basic characteristics are summarized in Table 1. 

Comorbidities recorded were psychiatric diseases (31.8%), mainly depression (16.9%), and addiction (12.7%), cardiovascular diseases (23.8%), while chronic renal diseases were recorded in 2.6% patients, and chronic liver diseases in 3.7%. Three patients were infected with SARS-CoV2 virus. In total, 214 patients (19.9%) had no associated comorbidities. Complete recovery was observed in 33% of patients, 62.9% developed one or more complications resolved before discharge, and 44 patients died during hospitalization (4.1%). The most frequently observed were CNS (13.2%), gastroenteric and cardiovascular complications (9.8%, and 8%, respectively).

Presence of diabetes was significantly correlated with mortality in acutely poisoned adults (*p* < 0.001). In addition, an altered mental status (GCS < 8), the PSS and the need of ICU therapy were correlated with mortality in both nondiabetic and diabetic patients (Table 1). We found no correlation between gender, poisoning intent, and body mass index (BMI) with mortality in acutely poisoned adults, regardless of diabetes status.

### 3.2. Admission Blood Glucose Level and Outcomes

**Figure 2:**
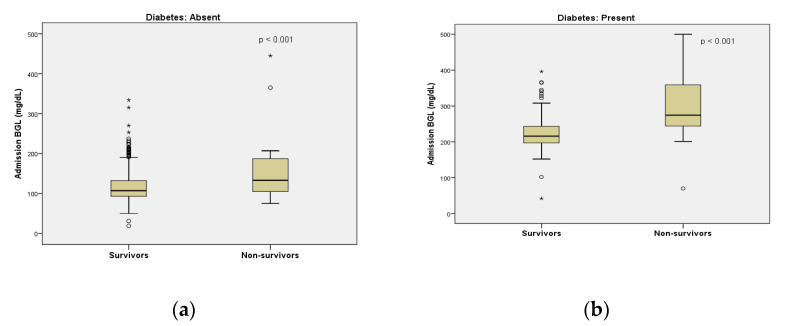
Box plot demonstrating the effect of admission blood glucose levels on mortality in patients without diabetes mellitus (**a**) and in patients with diabetes (**b**). Values are median and interquartile range; dots (°) represent outliers; * represent extreme values (shows non-normal distribution).

The admission BGL was not correlated with the BMI of the patients. In addition, it was not correlated with the time after exposure to a xenobiotic. BGL was significantly correlated with mortality both in nondiabetic and diabetic acutely poisoned patients (Table 1, Figure 2). The mortality rate in poisoned patients with hyperglycemia upon ED admission was significantly higher as compared with patients with a normal glucose level (65.9% vs. 15.9%, *p* < 0.001).

We found that BGL upon admission is significantly correlated with the poison involved (Table 2) in acutely poisoned nondiabetic patients. Higher BGL was recorded in pesticide poisoning compared with all other groups of poisons analyzed (*p* < 0.014). BGL was significantly higher after exposure to toxic alcohols and chemicals compared with drug poisoning (*p* < 0.001), caustic agent poisoning (*p* 0.006) and combination of toxin acute poisoning (*p* < 0.001). Acute poisoning with toxic gases resulted in higher BGL upon admission compared with poisoning involving drugs (*p* < 0.001), combination of toxins (*p* 0.001), and caustic agents (0.044). In addition, significant differences in BGL were recorded in acute poisoning with plant toxins, compared with drug poisoning (*p* 0.005) and combination of toxins (*p* 0.018) in nondiabetic adults. However, in diabetic patients, BGL was significantly higher in toxic alcohols and chemicals acute poisoning compared with poisoning with prescription drugs (*p* 0.02), combinations of toxins (0.001), and pesticides (0.024).

Univariate logistic regression revealed that the admission BGL was associated with mortality (OR = 1.015, 95% CI = 1.011–1.019, *p* < 0.001). Multivariate logistic regression (Table 3) confirmed that admission glucose level was a predictor of mortality (OR = 1.007, 95% CI = 1.002–1.013, *p* 0.005). There was no significant statistical influence of the CCI with regards of mortality in our cohort (Supplementary Table S1).

ROC curve analysis (Supplementary Figure S1) revealed that admission BGL had a good predictive value for in-hospital mortality (area under the curve (AUC) = 0.744, 95% CI = 0.648–0.841, *p* < 0.001). The cut-off value corresponding to the minimal false-negative and false-positive results for BGL was 104.5 mg/dL with 84% sensitivity, 41% specificity, 6% positive predictive value, 98% negative predictive value.

The admission BGL was significantly correlated with the need of ICU therapy (*p* < 0.001). Time spent in the ICU was significantly increased in patients with a moderate outcome (5.4 ± 4.2 days), and a poor outcome (7.5 ± 6.2 days) as opposed to patients with a good outcome (1.9 ± 0.1 days, *p* < 0.001). 

Patients with hyperglycemia (22%) and impaired glucose level (25.7%) upon admission developed significantly more complications during hospitalization, as opposed to patients having a normal glucose level and hypoglycemia upon presentation to the ED (17.9% and 1.4%, respectively, *p* < 0.001). BGL was correlated with the moderate and poor outcomes (Table 4). The admission BGL was significantly higher in patients with a moderate outcome (130.51 ± 51.1 mg/dL) and a poor outcome (215.05 ± 116.5 mg/dL) as opposed to patients with a good outcome of the poisoning (113.96 ± 36.77 mg/dL, *p* < 0.001). 

Univariate logistic regression revealed that the admission BGL was associated with in-hospital complications (OR = 1.010, 95% CI = 1.006–1.013, *p* < 0.001). Multivariate logistic regression confirmed that the admission BGL was a predictor of a moderate outcome (Table 4). ROC curve analysis revealed that ED admission glucose had acceptable predictive value for in-hospital complications (AUC = 0.618, 95% CI = 0.584–0.653, *p* < 0.001). 

### 3.3. Other Glycemic Parameters and Outcomes

When analyzing MGL, SD, CV, MAGE, and MAG with the toxin involved in the poisoning, we observed that in nondiabetic patients, only CV was significantly lower in plant toxin poisoning (0.11 ± 0.12%) as compared with poisoning with OTC medicines (0.18 ± 0.17%, *p* 0.034), pesticides (0.18 ± 0.15%, *p* 0.023), toxic gases (0.18 ± 0.18%, *p* 0.04), and with combination of toxins (0.16 ± 0.15%, *p* 0.039). In diabetic poisoned patients, MGL, SD and CV were also correlated with the poison type. MGL was significantly higher in diabetic patients poisoned with OTC medicines (222.25 ± 76.01 mg/dL), as opposed to patients poisoned with prescription drugs (131.62 ± 50.46 mg/dL, *p* 0.046), caustic agents (128.6 ± 37.95 mg/dL, *p* 0.05), combination of toxins (126.58 ± 39.96 mg/dL, *p* 0.042), and pesticides (126.15 ± 85.53 mg/dL, *p* 0.039). SD was significantly higher in diabetic patients poisoned with toxic alcohols and chemicals (31.07 ± 36.29 mg/dL), compared with poisoning involving combination of toxins (10.64 ± 15.33 mg/dL, *p* 0.038). CV was higher in diabetic patients diagnosed with toxic alcohols and chemicals acute poisoning (0.19 ± 0.18%, *p* 0.016) and prescription drugs overdose (0.17 ± 0.12%, *p* 0.043), versus patients poisoned with combination of toxins (0.07 ± 0.09%).

MGL, SD, CV, MAGE, and MAG were not significantly correlated with mortality. However, MAG was significantly higher in patients with a moderate outcome (24.59 ± 28.63 mg/dL/h), compared with patients with a good outcome (20.48 ± 27.3 mg/dL/h, *p* 0.035), irrespective of diabetes status. 

After univariate logistic regression, both MGL (OR = 1.007, 95% CI = 1.000–1.013, p 0.034) and CV (OR = 40.578, 95% CI = 1.349–1220.519, *p* 0.033) were predictive for in-hospital complications. (Supplementary Table S2). CCI was not correlated statistically significant with in-hospital complications in our cohort (Supplementary Table S3).

## 4. Discussion

In this cohort of unselected, prospectively followed nondiabetic and diabetic patients with acute poisoning within 12 h of exposure to a xenobiotic, several glycemic parameters during hospitalization were associated with the worst outcomes. While hypoglycemia is a common feature of overdose with sulphonylureas, insulin, ethanol, non-selective beta-blockers and paracetamol, hyperglycemia is not a common feature of poisonings [9,29]. Although acute poisonings represent an important cause of significant morbidity and mortality in patients admitted to the ED, the value of BGL on admission as an outcome predictor had been previously studied only in poisoning with methanol and pesticides [14,15,16,30,31]. Several glycemic parameters are associated with mortality and outcomes in different clinical situations, such as pneumonia, exacerbation of COPD, acute coronary syndromes, in cardiac arrest survivors, post-operatively, or in patients admitted in ICU for medical or surgical problems [3,4,5,8,12,32,33]. However, multiple glucose parameters were not studied in nondiabetic and diabetic patients acutely poisoned with xenobiotics.

Our study demonstrated that the admission BGL is important in predicting the outcomes of the hospitalized patients acutely poisoned with xenobiotics, regardless of diabetes status. Admission BGL was a predictor of mortality in acutely poisoned hospitalized patients. Our study revealed that the in-hospital mortality rate among patients with hyperglycemia upon ED admission was very high. With the exclusion of poisoned patients that received drugs influencing the glucose levels as part of the initial stabilization, we might conclude that hyperglycemia was induced by the stress of poisoning, which is an acute severe illness, inducing significant degrees of metabolic stress, and as a consequence, transient hyperglycemia [34]. Another explanation could be the toxin’s effect on the organs/systems involved in glucose homeostasis (i.e., liver, pancreas). The exact mechanism of the high predictive value of stress hyperglycemia on admission for adverse outcome in acute poisoning is not known. This could be due to gluconeogenesis, high adrenergic drive, or oxidative stress, similar to the mechanism encountered in other acute clinical situations [3,18].

In the current study population, the incidence of stress hyperglycemia in accidental and deliberate poisoning with undifferentiated xenobiotics was comparable with previous reports in self-poisoning with agents that do not produce hyper- or hypoglycemia [16], while the incidence of hypoglycemia was lower in our study. We found that BGL is correlated significantly with the type of poison, both in nondiabetic and diabetic patients. The incidence of hyperglycemia upon admission is high in patients poisoned with pesticides, toxic alcohols and chemicals, as well as toxic gases. In our cohort, OP pesticides represented the majority of pesticide poisoning recorded. It is known that OPs induce metabolic pathways in brain, skeletal muscles, and liver in favor of increased glucose production, and there is an involvement of oxidative/nitrosative stress. In addition, insulin resistance, disturbed insulin secretion, and pancreatitis are the consequences of OP exposure, which might explain the hyperglycemia recorded in this poisoning [31].

The high incidence of hyperglycemia upon admission in both nondiabetic and diabetic patients poisoned with a toxic alcohol that our study revealed can be explained, apart from stress-induced hyperglycemia, by the effect of methanol on the pancreas, acute pancreatitis being a recognized early complication in this toxicity [14]. Hyperglycemia was noticed in acute ethylene glycol (EG) poisoning, both in experimental studies and in clinical settings. EG poisoning can cause transient pancreatitis which results in reduction in serum insulin level; also an insulin resistance can be seen in association with acute renal failure which develops in EG poisoning within 24–72 h of exposure [35].

One of the original contributions of the present study is the effect that moderate glucose elevation upon admission has on mortality. In addition, we did not find a significant influence of CCI on short-term hospital mortality and morbidity in this cohort of acutely poisoned patients, similar to other studies involving acutely ill patients [36,37]. 

Complications and death were more often present in patients with hyperglycemia (defined as > 126 mg/dL) in a study which assessed deliberate self-poisoning in 345 non-diabetic patients, with a mortality rate of 0.6% [16]. Hyperglycemia is found to be associated with increased risk of infectious complications and septic shock, reduced immune response, dehydration and electrolyte imbalances and lethal multiple organ failure in traumatic and acute ischemic events [12,32,33,38]. In our study, hyperglycemia and impaired admission glucose were associated with multiple complications during hospitalization, and we proved that higher glucose levels upon admission had a predictive value for the development of in-hospital complications. 

Another original contribution of this study was to show that none of the glycemic parameters with a predictive value for mortality in other categories of medical or surgical patients [12,32,38] proved efficient in predicting mortality in acutely poisoned patients. Although CV is correlated with the poison type in nondiabetic intoxicated patients, and MGL, SD, and CV are correlated with the toxin involved in poisoned diabetic adults, only MGL and CV proved to be predictive for a moderate outcome in acute poisoning, while MAG is significantly higher in patients with this outcome, irrespective of diabetes presence. MAG was associated with adjusted hospital mortality in surgical patients but not in medical patients [38]. In medical ICU patients, only SD was independently associated with mortality [12]. High glucose variability by itself mediates harm by increasing oxidative stress, endothelial cell damage, mitochondrial damage, and coagulation activation [32]. Since continuous glucose sensors are not widely available in many countries, methods using intermittent glucose measurements, such as MAG, have been used instead of measures requiring continuous glucose monitoring [39]. 

Our study has several limitations. First, as a prospective single center study, the results may not be representative of all patients with acute poisoning with a xenobiotic. Thus, a multicenter study should be conducted to confirm our findings. Second, we did not have a standard protocol for monitoring the frequency of blood glucose levels in the first 24 h after admission. The relationship between blood glucose levels and the timing of a last meal before ED admission could not be assessed. However, most patients had at least 3-h delay between exposure to poison and admission to the hospital, with no food being consumed during this period. Third, the number of poisoned patients with DM was too small for conclusions to be drawn on the relation of glycemic variability and outcomes in this subgroup of patients. We did not systematically record HbA1c to be able to analyze the glycemic gap as part of the glycemic variability parameters in this cohort. Finally, there were wide 95% CIs for certain analyses, despite a significant p value, resulting from the relatively small sample size of each group, which implies a lower precision of the sample parameter, so a larger sample size is needed to replicate our results [40]. 

Despite these limitations, our study showed that there are significant relationships between glucose variability and short-term outcomes in both nondiabetic and diabetic poisoned adults. In addition, this is the first study to compare different glucose variability parameters in a cohort of patients acutely poisoned with xenobiotics.

## 5. Conclusions

This study reveals that several glycemic parameters assessed in acutely poisoned nondiabetic and diabetic patients with xenobiotics, hospitalized in a medical or ICU ward, are predictive for their outcomes. Thus, the admission blood glucose level is predictive for in-hospital mortality and could provide an early risk assessment tool for the patients acutely poisoned with xenobiotics. The mean glucose level and coefficient of variation of glucose after admission are predictive for in-hospital complications’ development and might be considered for use as a prognostic tool for short-term outcomes in acutely poisoned patients, hospitalized in a medical or ICU department. High glucose level upon admission in a stressful situation, such as acute poisoning, is correlated with the outcomes. It is useful for practitioners to benefit from this outcome predictor, easily available in the ED, when the patients are admitted within 12 h of exposure to xenobiotics. Whether intervention to prevent either pattern of changing glycemia would affect outcomes in this setting needs further studies.

## Figures and Tables

**Table 1:** Baselines characteristics of the cohort.

Parameter	Nondiabetic Poisoned Patients (*n* = 970)	*p*-Value *	Diabetic Poisoned Patients (*n* = 106)	*p*-Value
Age (years)	44 [32–60]	<0.001	57 [42–67.25]	0.083
CCI score (S/N, %)		0.757		0.896
CCI 0	96.8/3.2		-	
CCI 1–2	97.9/2.1		83.1/16.9	
CCI 3–4	98.1/1.9		85.2/14.8	
CCI ≥ 5	97.7/2.3		80/20	
Poison type (S/N, %)		0.422		0.021
Combination of poisons	26.8/0.8		13.2/0	
Drugs/medicines	38.2/0.7		29.2/2.8	
Non-pharmaceuticals	32.2/1.1		40.6/14.2	
GCS (S/N, %)		0.002		0.001
≥ 8	81.6/1.5		58.5/4.7	
< 8	15.7/1.1		24.5/12.3	
PSS (S/N, %)		<0.001		<0.001
Minor	44.3/0		25/0	
Moderate	42.6/30.8		53.4/11.1	
Severe	12.8/65.4		21.6/55.6	
Fatal	0.2/3.8		0/33.3	
SBP (mmHg)	125 [110–140]	0.018	135 [104–153.5]	<0.001
HR (bpm)	84 [73–100]	0.048	91 [75–114]	0.147
pH	7.39 [7.35–7.43]	0.837	7.37 [7.25–7.41]	<0.001
K+ (mmol/L)	4 [3.7–4.3]	0.984	3.9 [3.4–4.43]	<0.001
CRP (mg/dL)	0.37 [0.12–1.49]	<0.001	0.59 [0.15–1.92]	0.189
Hb (g/dL)	13.70 [12.5–14.9]	0.279	13.4 [12.4–14.53]	0.612
BGL (mg/dL)	109 [93–132]	<0.001	221.5 [200.5–266.25]	<0.001
MGL (mg/dL)	109 [94.58–136.25]	0.386	112.84 [94.92–140.19]	0.935
SD (mg/dL)	12.02 [4.51–29.16]	0.759	13.20 [4.95–27.93]	0.658
CV (%)	0.11 [0.04–0.24]	0.781	0.12 [0.05–0.21]	0.915
MAGE (mg/dL)	28 [7–94.25]	0.899	40 [9–117.75]	0.831
MAG (mg/dL/h)	13 [6–28]	0.532	13.55 [5.7–27.31]	0.696
Creatinine (mg/dL)	0.77 [0.69–0.90]	<0.001	0.83 [0.73–1.05]	<0.001
ALAT (U/L)	20 [14–32]	0.133	27 [17–48.5]	0.044
ICU therapy (S/N, %)		<0.001		<0.001
No	82.9/0.2		60/1.9	
Yes	14.4/2.5		22.9/15.2	
ICU hospitalization (days)	4 [3–6]	<0.001	5 [3–7.25]	0.631

Data are presented as median [25–75 percentile], or percentage; *, between survivors (S) and non-survivors (N); CCI, Charlson comorbidity index; GCS, Glasgow Coma Scale score; PSS, poisoning severity score; SBP, systolic blood pressure; HR, heart rate; CRP, C reactive protein; BGL, blood glucose level; MGL, mean glucose level; SD, standard deviation; CV, coefficient of glucose variation; MAGE, mean amplitude of glycemic excursions; MAG, mean absolute glucose change per hour; ALAT, alanine aminotransferase; ICU, intensive care unit.

**Table 2:** Correlation between admission BGL with type of poison involved in nondiabetic and diabetic poisoned adults.

Poison Involved	Nondiabetic Patients (*n* = 970)	*p*-Value	Diabetic Patients (*n* = 106)	*p-*Value
Prescription drugs	107.67 ± 25.899	<0.001	233.72 ± 52.949	0.066
Combination of poisons	110.54 ± 27.422		194.79 ± 61.481	
Pesticides	144.05 ± 54.999		227.75 ± 70.404	
Caustic agents	115.26 ± 33.349		244.80 ± 80.372	
Toxic alcohols and chemicals	130.86 ± 56.109		280.45 ± 90.574	
Toxic gases	127.77 ± 31.167		257.14 ± 57.389	
OTC	110.65 ± 24.245		231.00 ± 46.669	
Plant toxins	125.45 ± 28.346		203.33 ± 19.009	
Drugs of abuse	120.40 ± 33.721		228.33 ± 98.083	

Data are presented as mean ± standard deviation; OTC, over the counter medicines.

**Table 3:** Independent predictors of mortality identified with logistic regression analysis including initial glucose level and other statistically significant variables, which can easily be assessed at presentation.

Variable	Univariate Logistic Regression			Multivariate Logistic Regression		
	OR	95% CI	*p*-Value	OR	95% CI	*p*-Value
Age	1.065	1.033–1.098	<0.001	1.065	1.033–1.098	<0.001
GCS < 8	0.174	0.094–0.321	<0.001	2.774	0.933–8.244	0.066
CRP	1.066	1.023–1.111	0.003	0.992	0.933–1.055	0.804
BGL	1.015	1.011–1.019	<0.001	1.007	1.002–1.013	0.005
ICU therapy	0.019	0.007–0.054	<0.001	0.021	0.005–0.088	<0.001
Creatinine	1.650	1.230–2.212	0.001	1.176	0.813–1.699	0.389
Lactate	1.480	1.35–1.62	<0.001	1.349	1.199–1.517	<0.001

OR, odds ratio; CI, confidence interval; GCS, Glasgow Coma Scale score; CRP, C-reactive protein; BGL, admission blood glucose level; ICU, intensive care unit.

**Table 4:** The significant variables influencing the outcomes identified after multinomial logistic regression.

General Outcome ^a^		B	Std. Error	Wald	*p*-Value	OR	95% CI
Moderate	Age	0.009	0.005	3.666	0.056	1.009	1.000–1.018
	Admission BGL	0.005	0.002	5.451	0.020	1.005	1.001–1.009
	Creatinine	0.725	0.354	4.205	0.040	2.065	1.033–4.131
	Lactate	0.086	0.044	3.796	0.051	1.090	0.999–1.188
	GCS > 8	−1.689	0.426	15.695	0.000	0.185	0.080–0.426
	No ICU therapy	−1.259	0.433	8.448	0.004	0.284	0.121–0.664
Poor	Age	0.076	0.017	20.068	0.000	1.079	1.044–1.116
	Admission BGL	0.013	0.004	14.105	0.000	1.013	1.006–1.020
	Creatinine	0.854	0.390	4.802	0.028	2.348	1.094–5.040
	CV	6.758	3.653	3.422	0.064	860.937	0.669–1,107,985.854
	MAG	−0.048	0.026	3.310	0.069	0.954	0.906–1.004
	Lactate	0.385	0.074	26.759	0.000	1.469	1.270–1.700
	No ICU therapy	−5.220	0.866	36.349	0.000	0.005	0.001–0.030

^a.^ The reference category is: Good; BGL, blood glucose level; GCS, Glasgow Coma Scale score; ICU, intensive care unit; CV, coefficient of glucose variability; MAG, mean absolute glucose change per hour.

## Data Availability

Data is contained within the article or Supplementary Materials.

## Supplementary Materials

The following are available online at https://www.mdpi.com/2075-4418/11/2/361/s1, Table S1: Independent predictors of mortality identified with logistic regression analysis including initial glucose level and CCI, which can easily be assessed at presentation; Table S2: Variables predictive for in-hospital complications; Table S3: Variables predictive for in-hospital complications analyzed including CCI score; Figure S1. Receiver operating characteristic (ROC) curve for admission BGL.
