# Supplementary material for: Association of Multiple Glycemic Parameters at Hospital Admission with Mortality and Short-Term Outcomes in Acutely Poisoned Patients
Source: Diagnostics (Basel). 2021 Feb 20;11(2):361. doi: 10.3390/diagnostics11020361 (PMC7924603; doi:10.3390/diagnostics11020361)
Supplement: Supplementary file 1 [file diagnostics-11-00361-s001.pdf]

Table S1. Independent predictors of mortality identified with logistic regression analysis including initial glucose level and CCI, which can easily be assessed at presentation.

Variable	Univariate logistic regression			Multivariate logistic regression		
	OR	95%CI	*p*-Value	OR	95%CI	*p*-Value
Age	1.065	1.033-1.098	<0.001	1.063	1.031-1.096	<0.001
GCS < 8	0.174	0.094-0.321	<0.001	3.090	1.001-9.539	0.050
CRP	1.066	1.023-1.111	0.003	0.989	0.929-1.053	0.738
BGL	1.015	1.011-1.019	<0.001	1.008	1.002-1.014	0.006
ICU therapy	0.019	0.007-0.054	<0.001	0.021	0.005-0.088	<0.001
Creatinine	1.650	1.230-2.212	0.001	1.173	0.812-1.695	0.394
CCI 1-2	0.388	0.138-1.090	0.072	0.941	0.189-4.678	0.941
CCI 3-4	0.637	0.223-1.820	0.400	0.826	0.164-4.159	0.817
CCI ≥ 5	0.464	0.142-1.517	0.204	0.407	0.065-2.559	0.338
Lactate	1.480	1.35-1.62	<0.001	1.350	1.198-1.520	<0.001

CCI, Charlson comorbidity index score; OR, odds ratio; CI, confidence interval; GCS, Glasgow Coma Scale score; CRP, C-reactive protein; BGL, admission blood glucose level; ICU, intensive care unit.

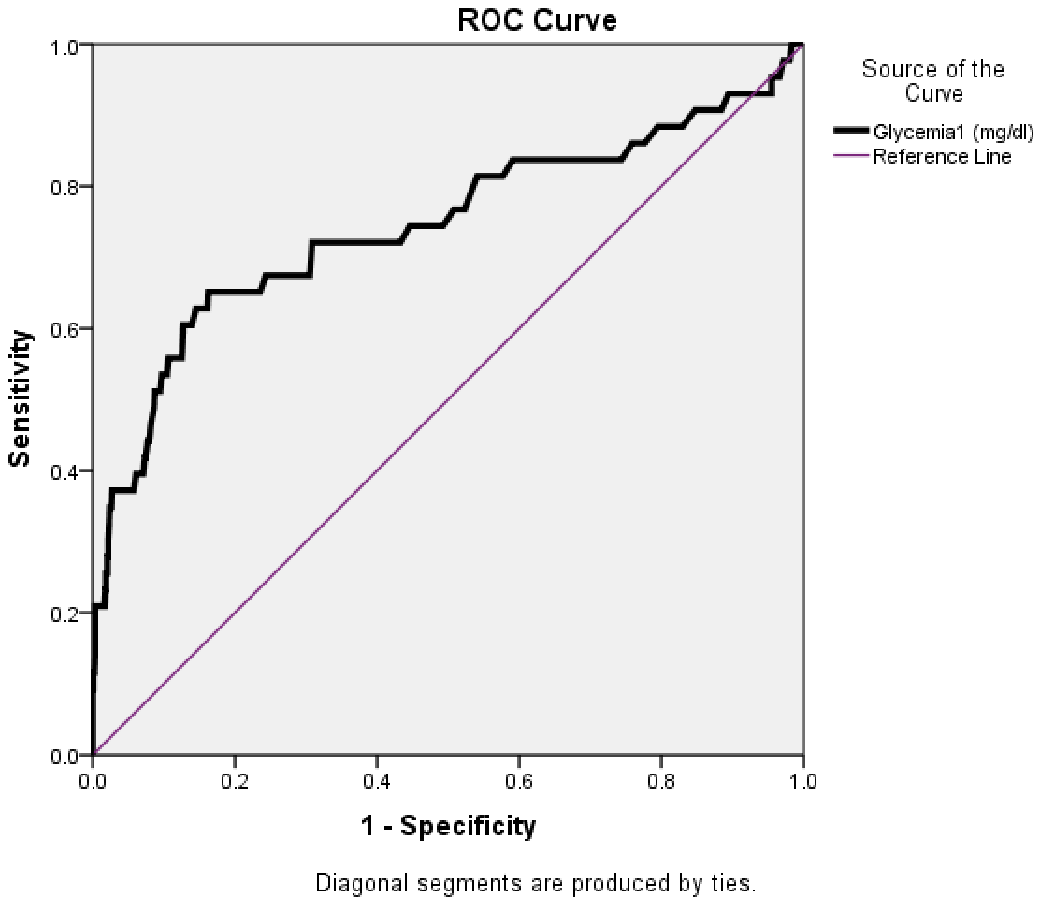

Figure S1. Receiver operating characteristic (ROC) curve for admission BGL.

Table S2. Variables predictive for in-hospital complications

		B	S.E.	Wald	df	Sig.	Exp(B)	95% C.I.for EXP(B)	
								Lower	Upper
Step 1[a]	Admission BGL	.006	.002	10.501	1	.001	1.006	1.002	1.010
	ICU therapy	-1.595	.417	14.619	1	.000	.203	.090	.460
	MGL	.007	.003	4.485	1	.034	1.007	1.000	1.013
	SD	-.025	.013	3.527	1	.060	.976	.951	1.001
	CV	3.703	1.737	4.547	1	.033	40.578	1.349	1220.519
	MAGE	-.003	.002	2.610	1	.106	.997	.994	1.001
	MAG	.012	.007	2.993	1	.084	1.013	.998	1.027
	Age	.010	.004	5.559	1	.018	1.010	1.002	1.018
	GCS < 8	-1.551	.398	15.206	1	.000	.212	.097	.462
	Constant	1.383	.623	4.925	1	.026	3.988		

[a.] Variable(s) entered on step 1: GCS < 1.

Table S3. Variables predictive for in-hospital complications analyzed including CCI score

		B	S.E.	Wald	df	Sig.	Exp(B)	95% C.I.for EXP(B)	
								Lower	Upper
Step 1[a]	Admission BGL	.007	.002	12.090	1	.001	1.007	1.003	1.011
	ICU therapy	-1.630	.414	15.468	1	.000	.196	.087	.442
	MGL	.007	.003	4.362	1	.037	1.007	1.000	1.013
	SD	-.026	.013	3.783	1	.052	.975	.950	1.000
	CV	3.857	1.742	4.900	1	.027	47.303	1.555	1438.542
	MAGE	-.003	.002	2.455	1	.117	.997	.994	1.001
	MAG	.012	.007	2.806	1	.094	1.012	.998	1.027
	CCI 1-2	-.721	.387	3.460	1	.063	.486	.228	1.039
	CCI 3-4	-.534	.398	1.800	1	.180	.586	.269	1.279
	CCI ≥ 5	-.765	.411	3.474	1	.062	.465	.208	1.040
	GCS < 8	-1.535	.396	15.065	1	.000	.215	.099	.468
	Constant	2.442	.714	11.686	1	.001	11.499		

a. Variable(s) entered on step 1: Coma.
